# A Bayesian functional approach to test models of life course epidemiology over continuous time

**DOI:** 10.1093/ije/dyad190

**Published:** 2024-01-10

**Authors:** Julien Bodelet, Cecilia Potente, Guillaume Blanc, Justin Chumbley, Hira Imeri, Scott Hofer, Kathleen Mullan Harris, Graciela Muniz-Terrera, Michael Shanahan

**Affiliations:** Jacobs Center for Productive Youth Development, University of Zurich, Zurich, Switzerland; Department of Laboratory Medicine and Pathology, Lausanne University Hospital, Lausanne, Switzerland; Jacobs Center for Productive Youth Development, University of Zurich, Zurich, Switzerland; Erasmus School of Health Policy and Management, Erasmus University Rotterdam, Rotterdam, The Netherlands; Jacobs Center for Productive Youth Development, University of Zurich, Zurich, Switzerland; Jacobs Center for Productive Youth Development, University of Zurich, Zurich, Switzerland; Biostatistics and Research Decision Sciences, MSD, Zurich, Switzerland; Jacobs Center for Productive Youth Development, University of Zurich, Zurich, Switzerland; Institute On Aging & Lifelong Health, University of Victoria, Victoria, BC, Canada; Carolina Population Center, University of North Carolina at Chapel Hill, Carolina Population Center, Chapel Hill, NC, USA; Center for Clinical Brain Sciences, University of Edinburgh, Edinburgh, UK; Ohio University Heritage College of Osteopathic Medicine, Ohio University, Athens, OH, USA; Jacobs Center for Productive Youth Development, University of Zurich, Zurich, Switzerland

**Keywords:** Life course models, Bayesian statistics, functional data analysis

## Abstract

**Background:**

Life course epidemiology examines associations between repeated measures of risk and health outcomes across different phases of life. Empirical research, however, is often based on discrete-time models that assume that sporadic measurement occasions fully capture underlying long-term continuous processes of risk.

**Methods:**

We propose (i) the functional relevant life course model (fRLM), which treats repeated, discrete measures of risk as unobserved continuous processes, and (ii) a testing procedure to assign probabilities that the data correspond to conceptual models of life course epidemiology (critical period, sensitive period and accumulation models). The performance of the fRLM is evaluated with simulations, and the approach is illustrated with empirical applications relating body mass index (BMI) to mRNA-seq signatures of chronic kidney disease, inflammation and breast cancer.

**Results:**

Simulations reveal that fRLM identifies the correct life course model with three to five repeated assessments of risk and 400 subjects. The empirical examples reveal that chronic kidney disease reflects a critical period process and inflammation and breast cancer likely reflect sensitive period mechanisms.

**Conclusions:**

The proposed fRLM treats repeated measures of risk as continuous processes and, under realistic data scenarios, the method provides accurate probabilities that the data correspond to commonly studied models of life course epidemiology. fRLM is implemented with publicly-available software.

Key Messages

- Models of life course epidemiology typically use discrete-time models whereby a limited number of repeated measures of risk are assumed to capture continuous exposure to risk.
- We propose a model that uses discrete data to test life course hypotheses over continuous time.
- Simulation studies reveal that the correct life course model can be identified with high probability with three to five repeated assessments of risk and 400 subjects.
- The method and software are illustrated with examples involving BMI trajectories from adolescence to mid-adulthood predicting mRNA-seq signatures of chronic health challenges.

## Introduction

Life course epidemiology often focuses on exposures to repeated risks and their consequences for health over many decades of life.^1^ Empirical studies are typically guided by three nested conceptual models: accumulation, which posits that all exposures to a repeated risk factor meaningfully predict the outcome; sensitive period, according to which more than one, but not all, exposures are predictive; and critical period, meaning that only one exposure matters.^2^ Although additional models are recognized,^3^ methodological research has focused on analytical strategies to determine which of these three models best corresponds to the observed data.^4–6^ The analytical task has been to: (i) estimate the association between exposure to risk and the outcome at each measurement occasion; and then (ii) decide which conceptual model is best supported by these estimates.

Madathil and colleagues proposed a relevant life course model (RLM) for continuously-scaled repeated exposures, measured in successive waves of a panel study to estimate weights associated with each measurement occasion and then select the most apt life course model based on these weights.^7^ First, for each subject *i*, the relevant life course exposure is conceptualized as the product between the continuously-scaled repeated risk $x_{t}$ and a weight reflecting its relevance at each of the measurement occasions. The outcome $y_{i}$ is then assumed to depend linearly on the sum of the relevant life exposure:

(1) $$y_{i}=\delta \sum_{t=1}^{T}x_{i,t}w_{t}\;+\;C_{i}^{\prime }\alpha \;+\;\epsilon_{i}.$$

where $w_{t}\geq 0$, $\sum_{t=1}^{T}w_{t}=1$, are weights, $C_{i}$ are covariates and $\epsilon_{i}$ random errors. The parameter δ represents the effect of the relevant life exposure $\sum_{t=1}^{T}x_{i,t}w_{t}$. Closely-spaced, discrete time points and *T* large, parametric shapes^8^ and non-parametric shapes^9–11^ for $w_{t}$ have previously been considered. In the RLM framework, the reference weights for the accumulation model refer to the case where $w_{t}=1/T$ for all *t*, the critical period model to the case where $w_{t}=1$ for one period and 0 for the others, and sensitive models to any other combinations. Second, Madathil and colleagues select the life course conceptual model based on the distance between the reference weights and the mean of the posterior distribution of weights.^12–14^

Drawing on the RLM, Chumbley and colleagues proposed a different strategy for deciding which life course model is most descriptive.^15^ The proposed method tests life course hypotheses by sequentially partitioning the simplex to identify the most credible ranking among the weights [e.g. that $w_{1}<w_{2}<w_{3}$ (a full ranking) or that $w_{1},w_{2}<w_{3}$ (a partial ranking)]. We refer to this method as the sequential partitioning test (SPT). SPT uses the greatest difference among the weights as test statistics to define regions of practical equivalence (ROPEs) for each of the three conceptual models.

The posterior probability of each model is then estimated by the fraction of posterior Markov Chain Monte Carlo (MCMC) samples falling into the corresponding ROPEs. For models not falling into the accumulation and critical period regions, post hoc decompositions then determine the most likely ranks for a sensitive period model.

Although discrete-time models such as the RLM correspond to the repeated assessments of risk that are often available in cohort studies, they assume that: (i) the association between risk and health involves discrete jumps corresponding to the time of measurement; (ii) the risk factors and health outcomes are measured at the moment corresponding to these jumps; and (iii) the measurement occasions include all relevant times of exposure to risk.^16^ Yet these assumptions may well be problematic in cases involving continuous processes of risk exposure. For example, addictive behaviours (such as consumption of tobacco or alcohol) are ongoing physiological assaults. Recently, substantial efforts have been made in the field of epidemiology to address these issues through the development of new functional approaches.^17–20^

**Figure 1.**
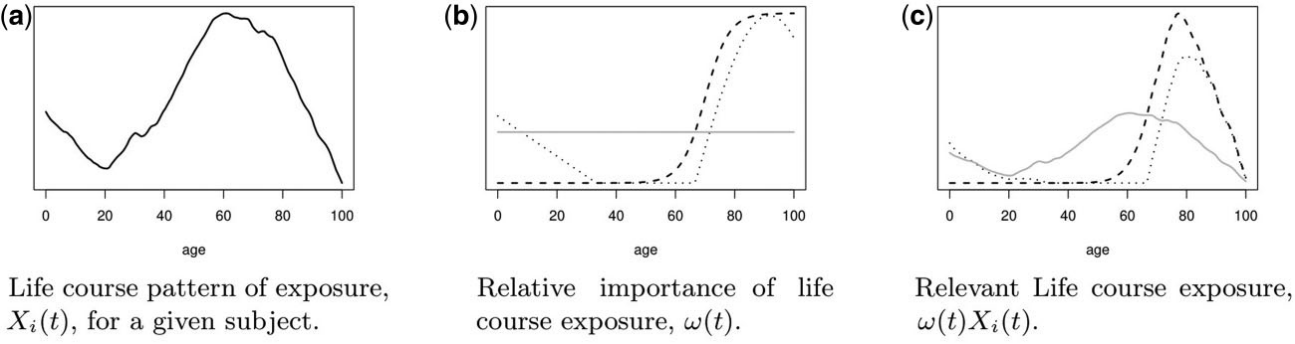
Simulated examples of continuous life course models representing under the accumulation (grey line), critical (dashed line) and sensitivity (dotted line) hypotheses

In this paper, we propose the functional relevant life course model (fRLM), an extension of the RLM, which takes into account that the observed risk data are only discrete measurements of an unobserved process changing continuously over time. Specifically, the fRLM assumes that the outcome depends on a weighted integral of the exposure as in:

(2) $$y_{i}=\alpha +\delta \int X_{i}(t)\omega (t)dt\;+\;C_{i}^{\prime }\alpha \;+\;\epsilon_{i},$$

where $X_{i}(t)$ are random functions observed at a finite number of discrete time locations, ω(t) is a continuous positive weight function and *t* now refers to the exact age. An example of the fRLM for the different life course conceptual models is provided in Figure 1, with the relative importance ω(t) based on discrete measures of risk. The number of measurements is allowed to vary across subjects (i.e. subject specific), and the fRLM is well suited to panel studies that begin with an age-heterogeneous group. Note that (i) can be also be seen as a particular case of (ii) when $X_{i}(t)$ are step functions. We consider a two-step estimation procedure of the fRLM and we show how to apply the SPT in order to test the different life course hypotheses.

This paper is structured as follows. We first describe the model and present the estimation method. We assess the performance of the model in identifying the most descriptive conceptual model of life course epidemiology given plausible data scenarios. Drawing on data from the National Longitudinal Study of Adolescent and Adult Health (Add Health), we then consider empirical examples that examine repeated assessments of body mass index (BMI) between ages 12 and 43, and gene expression (mRNA-seq) signatures representing the molecular underpinnings of chronic kidney disease (CKD), inflammation and breast cancer. The discussion subsequently highlights the advantages and drawbacks of our methodology. Additional simulation experiments are considered in the Supplementary Material (available as Supplementary data at *IJE* online) to compare the proposed method with alternative estimation procedures. fRLM and SPT are implemented in R with software available on GitHub.

## Methods

### The model

We consider data for which, for each subject indexed by i∈{1,2,…,n}, one observes a scalar outcome variable, $y_{i}$, along with repeated measurements of a time-varying risk exposure variable, $x_{i,j}$, observed at different time locations $t_{i,j}$, where $j\in \{1,2,\ldots ,N_{i}\}$. Note that both the number of measurement occasions $N_{i}$ and their specific timing $t_{i,j}$, may vary across subjects. We assume that the $x_{i,j}$ are discrete measurements of smooth functions of the continuous time, $X_{i}(t)$, specific to each subject. The functions $X_{i}(t)$ are not observed except at the specified time locations $t_{i,j}$, where we have $X_{i}(t_{i,j})=x_{i,j}$. The time *t* could be the age of subjects or the elapsed time after a lifetime event, for example, and lie in a specific time interval [*a*, *b*].

We assume that the data are generated by the following functional regression model:

(3) $$y_{i}=\delta \int_{a}^{b}X_{i}(t)\omega (t)dt+C_{i}^{\prime }\alpha +\epsilon_{i},$$

where the functional parameter ω(t) is a positive twice differentiable function that satisfies $\int_{a}^{b}\omega (t)dt=1$. The errors $\epsilon_{i}$ are assumed to be independently and identically normally distributed with mean 0 and variance $\sigma^{2}$; $C_{i}$ are *p*-dimensional non-functional covariates with $\alpha \in R^{p}$ being the corresponding covariate effects; and δ is a scalar parameter that represents the lifetime effect. The function ω(t) can be interpreted as a density, and the relative importance of a given period *T* can be computed as the integral $\int_{T}\omega (t)dt$.

### Estimation method

We provide an estimation procedure with two steps: Step 1, the prediction of each of the curves $X_{i}(t)$ based on the samples $x_{i,1},x_{i,2},\ldots ,x_{i,N_{i}}$; and then in Step 2, a Bayesian functional regression is estimated using the curves derived from Step 1, ${\hat{X}}_{i}(t)$.

In the first step, for predicting the individual curves, we make certain assumptions about their prior distribution. The random functions are assumed to be Gaussian Processes with different mean and covariance kernels for each subject, in order to allow for variability in the sample curves. The distribution of a Gaussian Process is fully specified by a mean function and covariance function (or covariance kernel). Specifically, we assume that $X_{i}(t)$ are Gaussian Processes with unknown mean $E[X_{i}(t)]=\mu_{i}(t)$ and covariance function $k_{i}(t,s)=\mathrm{Cov}(X_{i}(t),X_{i}(s))$. The parameterization of $k_{i}$ involves specific behaviours for the random functions, and we select the exponential covariance function, $k_{i}(t,s)=\nu_{i}^{2}\;\text{exp}\;\{-{\left(t-s\right)}^{2}/\kappa_{i}\}$, in order to ensure smooth patterns. Here $\nu_{i}$ and $\kappa_{i}$ are subject-specific hyperparameters called signal-variance and length-scale, respectively. For each subject, we assume a non-informative hyperprior for the hyperparameters and compute the maximum a posteriori (MAP) estimates. For each subject, the curves are predicted with the Gaussian Process regression method. Specifically, given realizations $x_{i,1},x_{i,2},\ldots ,x_{i,N_{i}}$, each curve is predicted at any time point *t*, by its conditional expectation, ${\hat{X}}_{i}(t)=E[X_{i}(t)|X_{i}(t_{i,1})=x_{i,1},X_{i}(t_{i,2})=x_{i,2},\ldots ,X_{i}(t_{i,N_{i}})=x_{i,N_{i}}]$. In the literature on functional regression, alternative methods have been proposed for estimating $X_{i}(t)$, such as the functional principal component analysis, used in the principal analysis by conditional estimation (PACE) method,^21^ and mixture of B-splines.^22^ In the Supplementary Material (available as Supplementary data at *IJE* online) we provide simulation experiments to compare the performance of these two estimation methods.

In the second step, we estimate a Bayesian functional regression on the predicted risk curves ${\hat{X}}_{i}(t)$, and prior distributions for the parameters have to be specified. Establishing a suitable prior for the functional parameter ω(t) requires care. Mixtures of (B-)splines are flexible, effective prior distributions used in non-parametric Bayesian statistics. Specifically, we model the functional parameter as a linear combination of B-splines, i.e. $\omega (t)=\sum_{l=1}^{L}\beta_{l}\phi_{l}(t)$. In this framework, ω(t) has to be positive with integral being one. To meet these two constraints, we used $\phi_{l}$ as density B-splines^23^ (i.e. rescaled B-splines satisfying $\int_{a}^{b}\phi (t)dt=1$), and constrain the parameters $\beta_{l}$ to belong to a simplex (i.e. we restrict them to be positive and to sum up to one, $\sum_{l=1}^{L}\beta_{l}$). The Dirichlet distribution is thus proposed, which is a natural distribution over the simplex and satisfies these constraints. A non-informative prior on the coefficient $\beta_{l}$ would be Dir(1,1,…,1). Finally, the Bayesian functional regression can then be estimated by computing the integrals $Z_{i,l}:=\int_{a}^{b}{\hat{X}}_{i}(t)\phi_{l}(t)dt$, and using them as regressors in a linear Bayesian regression model:

(4) $$y_{i}=\delta \sum_{l=1}^{L}Z_{il}\beta_{l}+C_{i}^{\prime }\alpha +e_{i},$$

where the $\beta_{l}$ have Dirichlet priors. The posterior distribution is obtained through MCMC simulations.

### Testing for models of life course epidemiology

The SPT procedure is then used to test which of the models of life course epidemiology best corresponds to the estimates: the accumulation, critical or sensitive period models.^15^ Although the SPT was proposed in the context of the linear RLM (1), the strategy applies to the fRLM as well. In the context of the fRLM, the user defines specific time periods of interest $T_{1},T_{2},\ldots ,T_{J}$, such that they form a partition of the unit interval [0, 1]. The specification of the time periods should be defined in the specific research context but might include, for example, age-based categories or processes before, during and after events (e.g. the pubertal transition). The user-defined time periods do not necessarily depend on the specifically-timed measurement occasions, which is a distinct advantage vis-à-vis the discrete RLM, according to which the time periods must coincide with the specific measurements.

The relative importance of the measurement occasions, $w_{j}$, for the period $T_{j}$ is then the integral of the weight function ω(t) over the period. That is, $w_{j}:=\int_{T_{j}}\omega (t)dt$. As $\left\{T_{j},j=1,2,\ldots ,J\right\}$ is a partition, $\left(w_{1},w_{2},\ldots ,w_{J}\right)$ belongs to a simplex. Thus, the SPT can be applied to $w_{j}$. The distribution of $w_{j}$ is obtained by integrating the functions ω(t) obtained across the MCMC samples.

## Evaluation of the fRLM with simulations

### Goals of the simulation

The fRLM and SPT are evaluated over a range of plausible data scenarios. Specifically, we consider the impact of the following on the ability of the model to recover simulated ground-truths:

- the underlying model of life course epidemiology (accumulation, and critical and sensitive period models);
- the sample size (n={100,400}); and
- the number of measurement occasions: a sparse scenario, (where $N_{i}$ is uniformly distributed over {3,4,5}), a moderately sparse scenario, ($N_{i}$ is uniformly distributed over {6,7,8}), and a scenario with completely observed trajectories (denoted by $N_{i}=\infty$). For the first two scenarios, we generated random observed time points by $t_{i,j}=\sum_{k=1}^{j}U_{i,k}/\sum_{k=1}^{N_{i}+1}U_{i,k}$, where $\left(U_{i,1},\ldots ,U_{i,N},U_{i,N+1}\right)$ are generated randomly from standard uniform variables for each simulation scheme and each subject. This allows us to obtain random time points satisfying $0<t_{i,1}<t_{i,2}<\ldots <t_{i,N_{i}}<1$.

We expect that with increasing sample size and number of observed time points, $N_{i}$, the performance of the estimates will improve.

### Parameters of the simulation

A functional regression model (3) was simulated with an intercept $C_{i}=1$ and errors from a normal distribution with variance $\sigma^{2}=2$, and δ=3 and α=1. The curves $X_{i}(t)$ were generated as Gaussian Processes with mean = 0 and variance = 1, and correlation kernel $k_{i}(t,s)=\text{exp}(-\kappa_{i}{\left(t-s\right)}^{2})$, where $\kappa_{i}$ was randomly generated from an exponential distribution with mean 1.

The data were generated from three different models:

- an accumulation model where ω(t)=1;
- a critical period model where $\omega (t)=\frac{10}{3(1+e^{-25(t-0.7)})}$; and
- a sensitive period model where
  (5) $$\omega (t)=\{\begin{array}{cc} 1.32(1-3t) & t\leq 1/3\\ 0 & 1/3<t\leq 2/3\\ 3.3\;\text{sin}(2\pi t-4\pi /3) & t>2/3. \end{array}$$

For the accumulation model, ω(t) is simply set to a constant. For the critical period model we parameterize ω(t) as a sigmoid function, which is used to yield a smooth transition between the non-critical period and the critical period. This allows ω(t) to meet the smoothness condition. For the sensitivity model, a general function is selected that is sparse over the interval [1/3,2/3]. The three functions are illustrated in Figure 1b.

### Numerical implementation

For the sparse and moderately sparse scenarios, the curves are estimated using maximum likelihood estimation for Gaussian processes. Regarding the choice of *L*, the selection of the number of B-splines bases is not crucial, as long as it is large enough to represent the complexity of the regression function.^24^

In this regard, taking into account the model complexity, the number of splines is set to L=4,6,7 for accumulation, critical and sensitive period models, respectively. We used the following prior distributions for the parameters:

$$\begin{array}{c} \beta ∼\mathrm{Dir}(1,\ldots ,1)\\ \delta ∼N(0,10)\\ \alpha ∼N(0,10)\\ \sigma ∼\;\text{log}\;N(0,1) \end{array}$$

Posterior distributions are obtained with MCMC simulations. To examine the properties of the fRLM to correctly identify the underlying life course model, the time interval is divided, for purposes of illustration, into three periods of equal lengths: $T_{1}=[0,1/3]$, $T_{2}=(1/3,2/3]$, and $T_{3}=(2/3,1]$. We then compute the posterior probability of the vector $\left(w_{1},w_{2},w_{3}\right)$, where $w_{j}=\int_{T_{j}}\omega (t)dt$. The analyst could change these based on theoretical considerations. Integrals are computed using Riemann approximations for each MCMC sample.

### Results of the simulation

Results of the simulation are reported in Table 1. We also report a summary of the convergence statistics and diagnostics in the Supplementary Material (available as Supplementary data at *IJE* online). The performance of the estimators is evaluated with the mean squared error between the estimates and the true underlying values for ω(t) and δ:

$$mse_{\omega }=\int_{0}^{1}{\left(\hat{\omega }(t)-\omega (t)\right)}^{2}dt,\;mse_{\delta }=|\hat{\delta }-\delta |.$$

**Table 1.** Performance metrics for the functional relevant life course model (fRLM) over 100 replications, median and median absolute deviation (MAD)

*n*	Setup	$mse_{\omega }$		$mse_{\delta }$		Pr(model|y)		$Pr(w_{2}<w_{1}<w_{3}|y)$	
Accumulation model									
100	3–5	0.051	(0.044)	0.116	(0.106)	0.514	(0.150)		
	6–8	0.041	(0.031)	0.073	(0.070)	0.666	(0.168)		
	∞	0.041	(0.030)	0.072	(0.063)	0.669	(0.156)		
400	3–5	0.034	(0.030)	0.092	(0.056)	0.940	(0.061)		
	6–8	0.020	(0.016)	0.044	(0.038)	0.992	(0.010)		
	∞	0.015	(0.013)	0.038	(0.035)	0.995	(0.006)		
Critical model									
100	3–5	0.194	(0.153)	0.076	(0.067)	0.674	(0.216)		
	6–8	0.186	(0.130)	0.063	(0.042)	0.760	(0.176)		
	∞	0.174	(0.117)	0.068	(0.043)	0.752	(0.172)		
400	3–5	0.125	(0.107)	0.069	(0.042)	0.921	(0.085)		
	6–8	0.070	(0.039)	0.027	(0.027)	0.973	(0.030)		
	∞	0.064	(0.030)	0.032	(0.026)	0.974	(0.029)		
Sensitive model									
100	3–5	0.192	(0.151)	0.158	(0.101)	0.724	(0.292)	0.749	(0.212)
	6–8	0.170	(0.145)	0.063	(0.061)	0.851	(0.172)	0.883	(0.129)
	∞	0.165	(0.126)	0.072	(0.050)	0.857	(0.176)	0.879	(0.132)
400	3–5	0.148	(0.069)	0.121	(0.057)	0.935	(0.090)	0.992	(0.012)
	6–8	0.128	(0.065)	0.043	(0.033)	0.972	(0.037)	0.999	(0.001)
	∞	0.116	(0.076)	0.030	(0.029)	0.980	(0.027)	1.000	(0.000)

The sample size is denoted by *n*. Setup indicates the number of measurement occasions for the simulation scenario: 3–5 for {3,4,5}, 6–8 for {6,7,8} and ∞ for completely observed trajectories. The mean squared errors for ω and δ are computed as $mse_{\omega }=\int_{0}^{1}{\left(\hat{\omega }(t)-\omega (t)\right)}^{2}dt$ and $mse_{\delta }=|\hat{\delta }-\delta |$, respectively. Following the sequential partitioning test (SPT) procedure, Pr(model|y) denotes the posterior probability of the ground-truth life course hypothesis (accumulation, critical or sensitive). For results indicating a sensitive model, $Pr(w_{1}<w_{2}<w_{3}|y)$ denotes the posterior probability of $w_{1}<w_{2}<w_{3}$ where $w_{j}=\int_{T_{j}}\hat{\omega }(t)dt$ and $T_{1}=[0,1/3]$, $T_{2}=[1/3,2/3]$, $T_{3}=[2/3,1]$ is a partition of (0, 1).

Following the SPT procedure, we report the posterior probability of the life course hypotheses for the omnibus test, Pr(model|y). For results indicating a sensitive model, we report the best sequence of nested sub-models of the sensitive model and their posterior probability.

Table 1 reveals, as expected, that performance of the fRLM improves with *n* but also with the average number of time points $N_{i}$. The $mse_{\omega }$ and $mse_{\delta }$ decrease, for each life course model, from a sample size of 100 and three to five measurement occasions to a sample of 400 with completely observed trajectories. The probabilities associated with identifying the correct life course model suggest that 100 cases are insufficient, but probabilities exceed.90 in all situations involving 400 cases. The correct identification of the full rank submodel (i.e. w2<w1<w3) is achieved with 400 cases and three to five measurement occasions (p=0.992).

## Empirical data example

We use data from the National Longitudinal Study of Adolescent to Adult Health (Add Health), which is a nationally representative longitudinal study of US adolescents in grades 7–12 in 1994–95 (age range 12–18) who were followed into adulthood over five waves of data collection.^25^ The BMI trajectory was measured from: Wave I (12–18 years), Wave II (14–20 years), Wave III (18–26 years), Wave IV (24–32 years) and Wave V (33–43 years). During Waves II, III, IV and V, field examiners collected height and weight measurements for each respondent. Self-reported height and weight were available for Waves I and V (measured height and weight were also collected during wave V). Wave V includes mRNA-seq abundance data from peripheral blood samples (for details of data collection protocol and the pre-processing of the data, see Shanahan *et al*.^26^).

We examine the association between BMI trajectories and three gene expression mRNA signatures: chronic kidney disease (CKD) (70 genes^27^), inflammation (751 genes^28^) and, for women only, breast cancer (BC) (44 genes).^29^^,^^30^ We used principal component analysis to reduce the dimensionality of each signature. The first principal component of each signature was used as the outcome.

We estimated Model (3) with Bayesian Hamilton Monte Carlo Markov Chains. For participant *i*, the assessments of BMI are denoted as $x_{t_{i,1}},x_{t_{i,2}},\ldots ,x_{t_{i,N_{i}}}$ performed at age $t_{i,1},t_{i,1},\ldots ,t_{i,N_{i}}$. Participants whose weights were missing for more than three waves were excluded from the analysis, and we thus have $3\leq N_{i}\leq 5$. The resulting sample sizes were n=3708 for CKD and Inflammation and n=2233 for Breast cancer. Covariates include biological sex, age at Wave V, number of hours fasting prior to blood draw, plate, use of anti-inflammatory medicines in the past 4 weeks, count of common clinical symptoms in the past 4 weeks (e.g. cold, fever, flu), count of common infectious and inflammatory diseases in the past 4 weeks (e.g. active infection, seasonal allergy) with correction for batch using ComBat.^31^ The BMI trajectories $X_{i}(t)$ were predicted using Gaussian Process regression for each individual. To estimate the functional model, we used L=7 density B-splines. The priors were the same as in the simulations.

For the testing procedure, we selected J=3 periods for illustrative purposes: $T_{1}=$ adolescence (age 12–18), $T_{2}=$ early-adulthood (age 19–29) and $T_{3}=$ mid-adulthood (age 30–40). The relative estimated importance of each period was computed by integrating the estimated weights, $w_{j}=|T_{j}{|}^{-1}\int_{T_{j}}\hat{\omega }(t)dt$. The ROPEs for the test statistics are selected as [0,0.2],(0.2,0.8),[0.8,1] for the accumulation, sensitive and critical models, respectively. The results of the omnibus test and post hoc decompositions are described in Tables 2 and 3. We also report a summary of the convergence statistics and diagnostics in the Supplementary Material (available as Supplementary data at *IJE* online).

**Table 2.** Omnibus test for posterior probability of the correct life course model

Signature	Accumulation	Sensitive	Critical
Chronic kidney disease	0	0.020	0.980
Inflammation	0.090	0.910	0.0005
Breast cancer	0.079	0.680	0.241

**Table 3.** Best sequence of partial rankings for the sensitive models for Inflammation and Breast cancer

Signature	Ranking	Probability
Inflammation	$w_{3}<w_{1}<w_{2}$	0.570
	$w_{3},w_{1}<w_{2}$	0.917
Breast cancer	$w_{1}<w_{2}<w_{3}$	0.781
	$w_{1},w_{2}<w_{3}$	0.936

Table 2 reports the Bayesian omnibus test of the three composite models as the posterior probability of the true composite model, i.e. Pr(model|y), where model∈{accumulation,critical, sensitive}. For the sensitive model, Table 3 reports the probabilities of the finest credible rankings.

**Figure 2.**
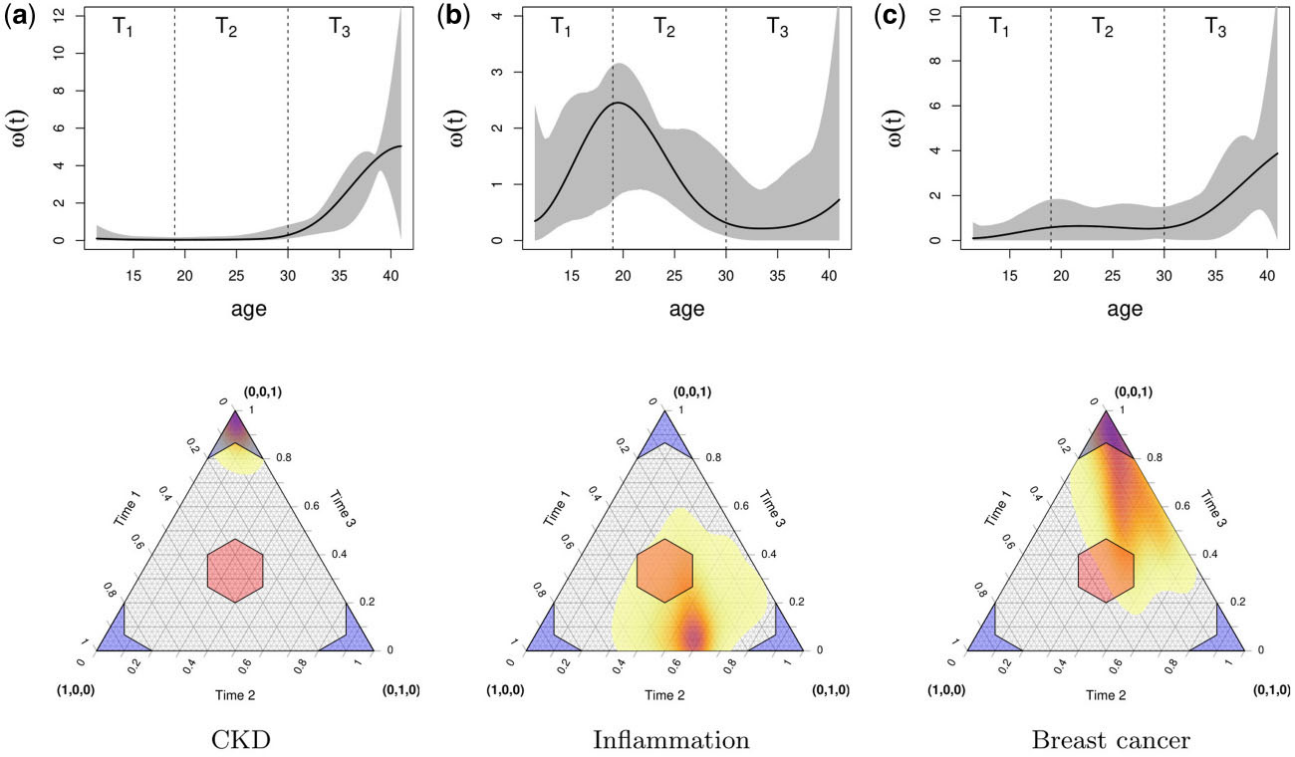
Estimation results for chronic kidney disease (CKD), inflammation and breast cancer gene signatures. Upper panels show the estimated relative importance $\hat{\omega }(t)$ (black lines), confidence bands (grey regions) and separation of time periods (dashed lines). Lower panels show the posterior distribution of the weights $w_{j}=|T_{j}{|}^{-1}\int_{T_{j}}\omega (t)dt$ as well as the regions of practical equivalenc(ROPEs) for the accumulation model (red region) and critical model (blue region)

For CKD, the omnibus test unambiguously identifies the critical period variant as the correct model (probability =0.98). Figure 2a indicates that Time period 3, middle adulthood, corresponds to the critical period. Nevertheless, because of the design of the study, this conclusion is tentative because middle-adulthood may not be critical (i.e. an age period of heightened vulnerability), but rather it reflects recency, meaning that the last measurement occasion, no matter what age range it might cover, would produce the same result.

The omnibus test for inflammation points to a sensitive period model (probability =0.91). The post hoc decomposition (Table 3) also reveals an unambiguous conclusion: that BMI in Time periods 1 and 2 is a more powerful predictor of inflammation than BMI in Time period 3 (probability =0.917). This conclusion is further supported by Figure 2b. Inflammation in middle adulthood is thus predicted by BMI in adolescence and early adulthood.

Table 2 reveals uncertainty, however, about the correct model for breast cancer, although the most warranted model is, once again, sensitive period (p=0.68). The post hoc decomposition shows that effect of BMI is greatest at Time 3 and the partial ranking of 1,2|3 is most supported (probability =0.936). The plotted $\hat{\omega }(t)$ in Figure 2c may suggest a critical period for Time 3, but the accompanying ternary plot shows considerable dispersion of the posterior distribution of weights beyond the ROPE. Thus, the breast cancer signature reflects BMI in middle adulthood, but the effects associated with adolescence and young adulthood are not negligible.

**Figure 3.**
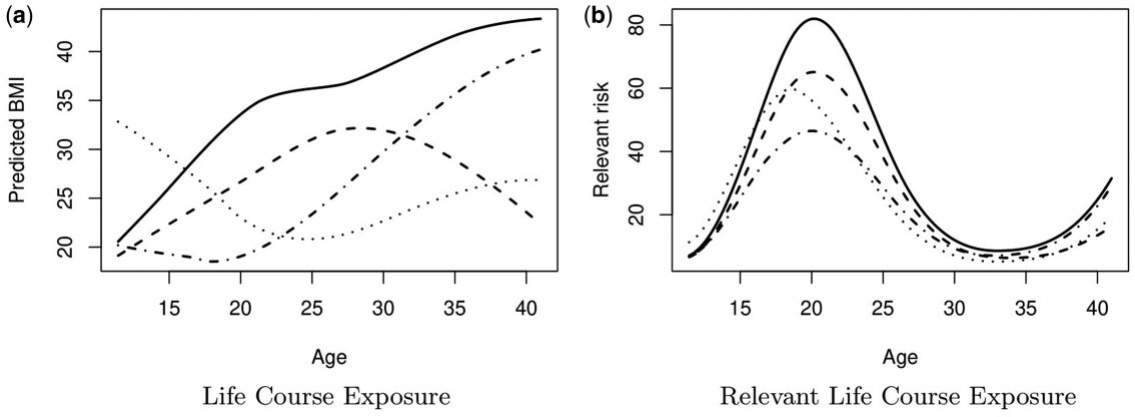
For four randomly selected subjects, predicted pattern of the body mass index (BMI),${\hat{X}}_{i}(t)$, and estimated relevant life course exposure, ${\hat{X}}_{i}(t)*\hat{\omega }(t)$, for the Inflammation gene signature

Finally, Figure 3 illustrates the patterns of BMI observed for four people and reveals considerable diversity in BMI trajectories: two individuals experienced precipitous increases in BMI, but the other two people experienced positive and negative fluctuations. The relative importance of BMI for inflammation is shown in Figure 2b, and Figure 3b shows the relevant exposure, which is the product of the BMI trajectories and $\hat{\omega }(t)$ in (3). The relevant exposure shows relatively similar patterns, i.e. a bimodal configuration. However some people exhibit much higher relevant risk than others, depending on the shape of their BMI trajectories.

## Discussion

We propose the functional relevant life course model (fRLM), which considers discrete, sparse measurements as unobserved processes occurring in continuous time. This analytical goal is appropriate when the risk factors being studied reflect continuous processes (e.g. substance use, poverty or income trajectories, blood glucose). The fRLM defines the total lifetime exposure to risk as an integral (2) according to which exposures are assumed to be unobserved smooth functions. Because *t* refers to the exact age of the person, the fRLM is best suited to panel studies that begin with an age-heterogeneous group, although the model can also be applied to birth cohort studies. We also test life course hypotheses by applying Chumbley *et al.*’s SPT procedure^15^ to our framework.

Simulations show that the performance of the fRLM improves with the number of repeated measurement occasions as expected, and the method is able to identify the correct life course model when n=400 at least, even for very sparse designs with three repeated measurements per person. Finally, the method is illustrated with three instructive empirical examples that examine the relationship between BMI trajectories from adolescence to middle adulthood, and mRNA-seq expression signatures for chronic kidney disease, inflammation and breast cancer.

Note that the proposed model extends the RLM but also differs from approaches^9–11^ that consider non-parametric estimation of the weights by regarding the observations as realizations of a continuous underlying process.

The closest model may be in the context of survival analysis,^19^ where a functional regression with a weight function satisfies ∫ω(t)dt=1 but this is allowed to be negative. The implementation is frequentist and is performed by first estimating β(t)=δω(t) and then identifying ω(t) by rescaling. The advantage of the present Bayesian implementation of the fRLM model is that it flexibly constrains parameters (i.e. the weight function is constrained to belong to a set of distributions by defining the appropriate prior distribution on the B-splines coefficients, the Dirichlet prior distribution). Prior models also consider densely, regularly spaced time points, whereas the fRLM allows for sparse and irregularly spaced time points. In this way the time index *t* need not correspond to the timing of measurement occasions, and is allowed to represent meaningful milestones based on the exact age of the subjects. Also, the model uses all available data and thus avoids limitations of methods for missing data. Finally, as discussed in the Supplementary Material, other methods can be used to estimate the fRLM.^21^^,^^22^

Franklin and their colleagues’ review of suicidal behaviours notes several requisites for a successful empirical study of risk,^32^ which represent strategic opportunities to extend the fRLM. First, the fRLM can accommodate multiple risk factors as a straightforward additive functional linear model, and interactions among different risks are also possible. Second, repeated assessments can also be modelled in dynamic terms, implemented with, for example, a function-on-function regression. Third, improvements in efficiency can be made by considering other processes (e.g. log-Gaussian process for positive data). Fourth, empirical studies of risk offer the promise of an increasingly personalized approach to health by providing people with a risk score, but such scores do not reflect the changing nature of risk across life [e.g. the Framingham risk score^33^ and the CAIDE (Cardiovascular Risk Factors, Aging, and Incidence of Dementia) score to predict dementia^34^]. The fRLM offers a method by which risk scores could reflect the changing nature of risk across the life course by, for example, reflecting the estimated relevant life course exposure, ${\hat{X}}_{i}(t)\hat{\omega }(t)$.

Nevertheless, the fRLM has several limitations. First, the risk exposure is modelled as a Gaussian Process, which excludes modelling of data with binary or discretely-scaled risk exposures. Thus, the health outcome and repeated risk factor must be continuously scaled, which rules out, for example, the study of caseness defined by clinical cut-offs. Second, the fRLM can not test chain-of-risk models (e.g. a Markov autoregressive model with an earlier risk factor predicting its later value, which in turn predicts the outcome). Chain-of-risk models are intrinsically discrete-time, however, in contrast to the fRLM’s depiction of risk as a continuous process. Finally, although we develop a broad framework for continuous risk exposure, some efficiency could be gained by setting priors that are more specific to the risk. For example, BMI is always positive, so it may improve the inference to set a positive prior distribution.

Despite these limitations, the fRLM offers a method by which discrete data can be used to model the experience of risk across many decades of life as a continuous process. Particularly in the context of life course epidemiology, many risks are chronic and thus the focus on continuous process is likely more realistic than discrete-time models.

A package including all the functions to perform the analyses is included on GitHub at the following address: [https://github.com/jbodelet/fRLM]. We also provide the simulations for reproducibility.

## Ethics approval

Add Health Study protocols were approved by the institutional review board at the University of North Carolina, approval #13–1946.

## Supplementary Material

dyad190_Supplementary_Data

## Data Availability

Add Health data are available through a restricted data-use contract [https://addhealth.cpc.unc.edu/data/#restricted-use].
