# Supplementary material for: A Bayesian functional approach to test models of life course epidemiology over continuous time
Source: Int J Epidemiol. 2024 Jan 10;53(1):dyad190. doi: 10.1093/ije/dyad190 (PMC10859158; doi:10.1093/ije/dyad190)
Supplement: dyad190_Supplementary_Data [file dyad190_supplementary_data.pdf]

Supplementary material: Bayesian functional approach to test models of life course epidemiology over continuous time

Julien Bodelet[1,2,*], Cecilia Potente[1,3], Guillaume Blanc[1], Justin Chumbley[1], Hira Imeri[1], Scott Hofer[4], Kathleen Mullan Harris[5], Graciela Muniz Terrera[6,7], and Michael Shanahan[1]

[1]Jacobs Center for Productive Youth Development, University of Zurich, Zurich Switzerland
[2]Lausanne University Hospital, Lausanne, Switzerland
[3]Erasmus University Rotterdam, Rotterdam, Netherlands
[4]Department of Psychology, University of Victoria, Victoria, Canada
[5]University of North Carolina at Chapel Hill, Carolina Population Center, Chapel Hill, NC, USA
[6]Center for Clinical Brain Sciences, University of Edinburgh, Edinburgh, UK
[7]Ohio University Heritage College of Osteopathic Medicine, Ohio University, Athens, OH, USA

[*]Corresponding author. Jacobs Center for Productive Youth Development, University of Zurich, Andreastrasse 15, Zurich CH8050, Switzerland. E-mail: julien.bodelet@chuv.ch

1 Complementary simulations

1.1 Simulation design

We further assess the proposed method, which estimates curves as Gaussian Processes (hereafter, GP), with additional simulations and provide comparisons approaches for estimating the functional curves, including the Principal Analysis by Conditional Estimation (PACE) method[1], the Bayesian implementation of the Penalized Functional Regression (PFR)[2], as well as with the RLM[3], which is the discrete version of the fRLM. The PACE and PFR methods are two mainstream methods in functional regression for longitudinal data[4,5] and we adapt them to estimate the fRLM. The PFR method uses B-splines to model the curves, and the PACE method performs a functional regression on curves estimated with functional PCA.

We consider a standard scenario where $\omega(t) = \cos(3\pi t + \pi/4) + 1$ is non-sparse. Errors are from a normal distribution with variance $\sigma^2 = 1$. Parameters are set to $\delta = 3$, $\alpha = 1$ with $C_i = 1$. Curves $X_i(t)$ were generated as random trigonometric functions,

$$X_i(t) = \sum_{m=1}^{10} a_m \cos(mt\pi/2) + b_m \sin(mt\pi/2),$$

where $a_m \sim b_m \sim \mathcal{N}(0, 1/m^2)$.

We consider different sample sizes ($n \in \{100, 400, 800\}$) and again a different number of measurement occasions: a sparse scenario, ($N_i \in \{3, 4, 5\}$), and a moderately sparse scenario, ($N_i \in \{6, 7, 8\}$). We generated random observed time points by $t_{i,j}$ as in the main simulation, but we restricted to the space such that there is at least one $t_{i,j}$ in each bin $[0, 1/3], (1/3, 2/3], (2/3, 1]$, for each i, in order to permit the comparison with the discrete RLM.

1.2 Numerical implementation of competing methods

For the PFR, PACE, and GP methods, we use the same prior distribution for the Bayesian functional regression step, that is:

$$\begin{aligned}
\boldsymbol{\beta} &\sim Dir(1, ..., 1)\\
\delta &\sim \mathcal{N}(0, 10)\\
\alpha &\sim \mathcal{N}(0, 10)\\
\sigma &\sim \log \mathcal{N}(0, 1)
\end{aligned}$$

For all approaches, we selected $L = 9$ B-splines basis function in order to balance flexibility of the approximations and computational burden.

For the PFR, we follow their approach and approximate the curves with $K = 9$ B-splines bases functions. For the PACE method, we used 15 functional Principal Components computed on a regular grid of size 150.

Finally, we provide comparisons with a discrete RLM with three periods. We aim to mimic the common approach in the literature when applying discrete models to longitudinal data with irregularly spaced observations. We divided the time interval $[0, 1]$ into three periods of equal lengths: $T_1 = [0, 1/3]$, $T_2 = (1/3, 2/3]$, and $T_3 = (2/3, 1]$. We average the observations collected on each interval and use them as a time varying exposure, that is we use the model

$$y_i = \delta \sum_{t=1}^{3} w_t x_{t,i} + C_i' \alpha + \epsilon_i$$

where $x_{t,i}$ is the average of the observations on interval T_t.

Table S1: Performance metrics (median and MAD) over 100 replications for different estimation methods: GP, PFR, PACE and RLM.

n	Setup	method	mse_ω		mse_δ	
100	3-5	GP	0.329	(0.093)	0.006	(0.008)
		PFR	0.260	(0.087)	0.157	(0.132)
		PACE	0.504	(0.102)	0.013	(0.019)
		RLM			0.029	(0.026)
	6-8	GP	0.218	(0.060)	0.002	(0.003)
		PFR	0.205	(0.075)	0.030	(0.030)
		PACE	0.469	(0.222)	0.010	(0.014)
		RLM			0.011	(0.014)
400	3-5	GP	0.275	(0.086)	0.010	(0.010)
		PFR	0.177	(0.081)	0.188	(0.073)
		PACE	0.515	(0.200)	0.006	(0.008)
		RLM			0.029	(0.017)
	6-8	GP	0.121	(0.036)	0.001	(0.001)
		PFR	0.192	(0.049)	0.025	(0.016)
		PACE	0.483	(0.329)	0.003	(0.004)
		RLM			0.015	(0.008)
800	3-5	GP	0.248	(0.070)	0.010	(0.006)
		PFR	0.195	(0.050)	0.182	(0.069)
		PACE	0.908	(0.710)	0.005	(0.007)
		RLM			0.031	(0.012)
	6-8	GP	0.114	(0.015)	0.00	(0.001)
		PFR	0.215	(0.027)	0.022	(0.010)
		PACE	0.398	(0.178)	0.001	(0.002)
		RLM			0.014	(0.006)

Table S2: Summary statistics (median and MAD) of the Mean squared errors of the estimated curves for $n = 800$.

Setup	method	mse_X	
3-5	GP	0.427	(0.021)
	PFR	0.544	(0.042)
	PACE	0.556	(0.070)
6-8	GP	0.116	(0.007)
	PFR	0.211	(0.012)
	PACE	0.272	(0.050)

1.3 Results

We performed 100 Monte Carlo runs for each scenario and used 4 chains of 500 iterations with 250 as warmup. For each method, we computed the mean square error for δ and ω when available and report them in Table S1. We also report in Table S2 the mean integrated squared error of $X_i(t)$,

$$mse_X = \frac{1}{n}\sum_{i=1}^{n}\int_0^1 (X_i(t) - \hat{X}_i(t))^2 dt,$$

where the integral is approximated with Riemann sums over a grid of length 150. Results were, as expected, very similar across all n and thus reported only for the largest n. Gaussian Processes are shown to outperform both PFR and the PACE method for both settings (very sparse and moderately sparse).

From the results shown in Table S1, we see that GP and PFR methods outperform the PACE method regarding $\omega(t)$. Regarding $\omega(t)$, the GP approach appears more advantageous in the $6-8$ setting and PFR for the $3-5$ setting. However, regarding δ, our approach outperforms the other models and the PFR method appeared to be the worst in all scenarios. Therefore, modeling curves with Gaussian Process methods appears to be superior for sparse data with irregularly spaced observations. This is likely to stem from the ability of the Gaussian Processes to better estimate the functional curves in sparse designs[6].

2 Convergence diagnostics

We provide a summary of the convergence diagnostics for the Hamiltonian Monte Carlo (HMC) implementation for Bayesian estimation in the simulations and the data analysis. This includes quantitative statistics: the Gelman-Rubin statistics[7] (or Rhat), effective sample size (ESS bulk and tail), which indicates the number of independent draws in the sample[8], and the proportion of number of divergent iterations after warmup (i.e. ratio of divergent iterations to total iteration after warmup). These statistics are reported for δ and ω and are provided in Tables S3, S4 and S5 for Simulation 1, the data analysis and Simulation 2 respectively.

The Rhat is a statistical measure used to determine if the chains are well mixed. Rhat should ideally be close to 1 and Rhat values greater than 1.2 suggest a lack of convergence. In our study, all parameters had Rhat values between 1.00 and 1.15, suggesting convergence for our chains. The effective sample size (ESS) is a measure that considers both the number of iterations and the autocorrelation between iterations. A higher ESS generally suggests better chain mixing, and is especially important in HMC, where autocorrelation can be substantial. ESS was always higher than 100, which is usually considered sufficient. The proportion divergent was always close to 0. For the data analyses, over 8000 total iterations, there was 14, 50, 46 divergent iterations for CKD, Inflammation, and Breast Cancer respectively. In addition, we report the traceplots for δ for each disease signature in Figure S1. Traceplots show that the chains are well mixed, indicating convergence.

Table S3: Simulation study 1: median of the convergence statistics.

n	Setup	δ			ω			Div
		Rhat	Bulk_ESS	Tail_ESS	Rhat	Bulk_ESS	Tail_ESS	
	Accumulation model							
100	3-5	1.01	564	543	1.01	396	339	0.00
100	6-8	1.01	552	554	1.01	372	328	0.00
100	∞	1.01	562	569	1.01	377	321	0.00
400	3-5	1.01	484	501	1.02	206	247	0.00
400	6-8	1.01	530	512	1.02	201	228	0.00
400	∞	1.01	517	510	1.02	193	235	0.00
	Critical model							
100	3-5	1.01	492	472	1.01	327	276	0.00
100	6-8	1.01	430	430	1.01	256	239	0.00
100	∞	1.01	443	436	1.01	253	247	0.00
400	3-5	1.01	464	432	1.01	252	240	0.00
400	6-8	1.01	420	382	1.01	278	243	0.00
400	∞	1.01	419	403	1.01	303	258	0.00
	Sensitive model							
100	3-5	1.01	484	513	1.01	361	308	0.01
100	6-8	1.01	500	512	1.01	347	287	0.01
100	∞	1.01	488	500	1.01	316	278	0.01
400	3-5	1.01	435	482	1.02	277	271	0.01
400	6-8	1.01	452	491	1.02	222	241	0.01
400	∞	1.01	405	460	1.03	200	215	0.01

Table S4: Data analysis: median of the convergence statistics for each parameter. For the covariates parameters(alpha) we report the median over all coefficient.

coeff	Rhat	Bulk_ESS	Tail_ESS
CKD			
delta	1.00	5437	3071
beta[1]	1.00	2249	1821
beta[2]	1.00	1931	1360
beta[3]	1.00	1795	988
beta[4]	1.00	2211	1854
beta[5]	1.00	1913	1709
beta[6]	1.00	1396	1096
beta[7]	1.00	1483	1091
sigma	1.00	6065	3137
alpha	1.00	1436	1668
Inflammation			
delta	1.00	2599	2774
beta[1]	1.00	1321	1092
beta[2]	1.00	1739	1572
beta[3]	1.00	793	250
beta[4]	1.00	1095	442
beta[5]	1.00	2003	1151
beta[6]	1.00	2687	2266
beta[7]	1.00	2415	1864
sigma	1.00	4058	2743
alpha	1.02	920	1613
Breast cancer			
delta	1.00	4353	3032
beta[1]	1.00	1828	1254
beta[2]	1.00	1947	1329
beta[3]	1.00	2121	1442
beta[4]	1.00	1915	1337
beta[5]	1.00	2357	1769
beta[6]	1.00	3250	2441
beta[7]	1.00	3577	2320
sigma	1.00	5354	3087
alpha	1.01	1803	1947

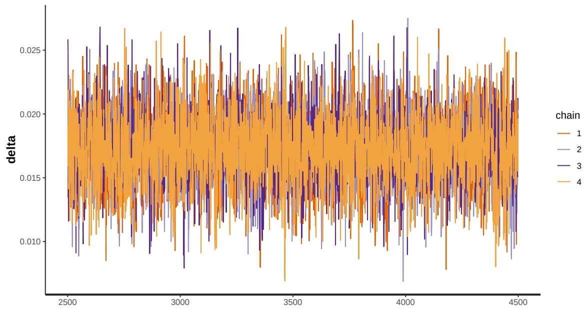

(a) CKD

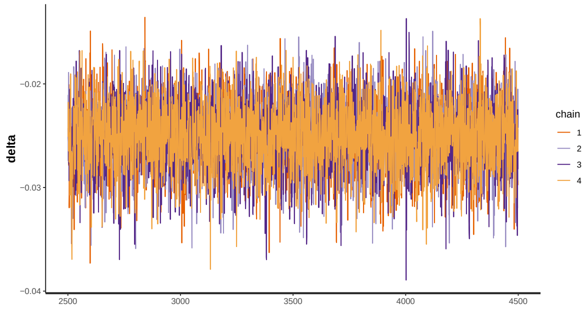

(b) Inflammation

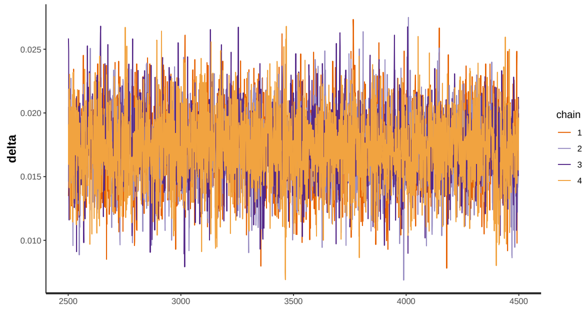

(c) Breast cancer

Figure S1: Data analysis: traceplot for the parameter δ.

Table S5: Simulation study 2: median of the convergence statistics.

n	Setup	method	δ				ω				Div
			Rhat	n_eff	Bulk_ESS	Tail_ESS	Rhat	n_eff	Bulk_ESS	Tail_ESS	
100	3-5	GP	1.010	269	282	275	1.030	192	151	164	0.030
		PACE	1.010	287	296	285	1.010	327	227	197	0.030
		RLM	1.010	564	584	377					0.000
		PFR	1.080	114	124	144	1.070	122	132	152	0.090
	6-8	GP	1.010	255	272	264	1.050	135	123	155	0.020
		PACE	1.010	259	282	298	1.020	255	183	183	0.030
		RLM	1.010	540	556	378					0.000
		PFR	1.020	150	162	289	1.030	196	184	218	0.040
400	3-5	GP	1.030	166	178	245	1.050	130	108	133	0.030
		PACE	1.020	154	173	225	1.030	234	161	172	0.030
		RLM	1.000	580	605	392					0.000
		PFR	1.150	110	120	154	1.090	77	81	140	0.080
	6-8	GP	1.020	231	242	257	1.050	123	112	125	0.010
		PACE	1.030	126	158	227	1.050	145	112	145	0.030
		RLM	1.010	576	585	381					0.000
		PFR	1.010	170	198	317	1.020	264	236	260	0.030
800	3-5	GP	1.020	186	199	222	1.050	126	112	132	0.030
		PACE	1.040	100	118	194	1.050	175	116	151	0.030
		RLM	1.010	564	594	381					0.000
		PFR	1.120	112	114	151	1.130	156	164	110	0.090
	6-8	GP	1.020	196	210	232	1.050	106	92	118	0.020
		PACE	1.030	156	176	214	1.050	125	102	123	0.040
		RLM	1.010	563	580	384					0.000
		PFR	1.010	245	276	332	1.020	268	235	267	0.030

3 Simulation based calibration

To validate our Bayesian model we used Simulation Based Calibration (SBC), which uses the self-recovering property of Bayesian models[9,10]. For this purpose we used the R package SBC[11]. We used 100 simulations of a simple fRLM model with $L = 4$ and an intercept. If the algorithm is correctly calibrated, SBC rank statistics should be uniformly distributed. Results are shown in Figure S2 and S3 and suggest uniformly distributed rank statistics.

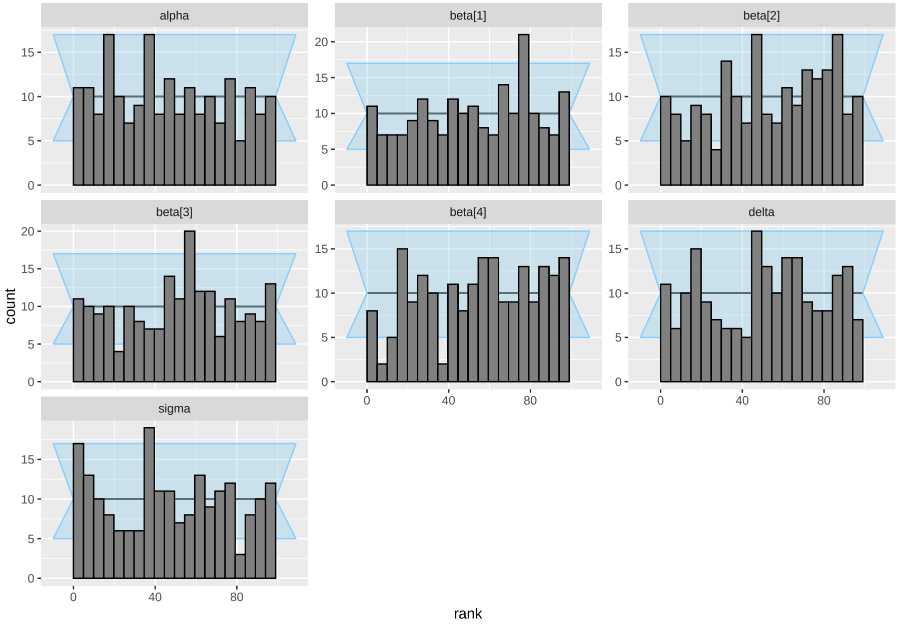

Figure S2: The distribution of SBC ranks.

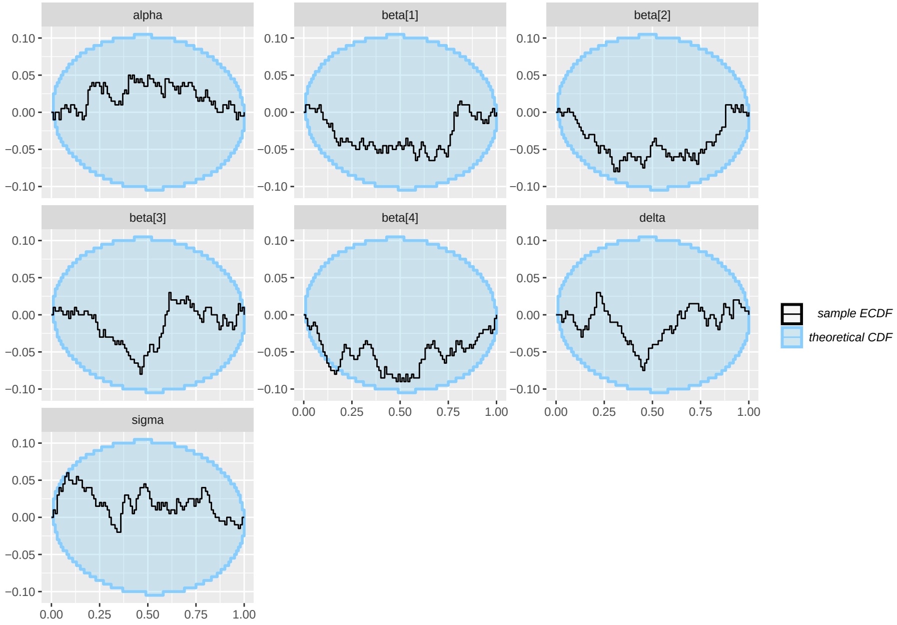

Figure S3: ECDF difference plot

References

[1] Yao F, Müller HG, Wang JL. Functional linear regression analysis for longitudinal data. Ann Stat. 2005;33:2873-903.

[2] Goldsmith J, Bobb J, Crainiceanu CM, Caffo B, Reich D. Penalized functional regression. J Comput Graph Stat. 2011;20:830-51.

[3] Madathil S, Joseph L, Hardy R, Rousseau MC, Nicolau B. A Bayesian approach to investigate life course hypotheses involving continuous exposures. Int J Epidemiol. 2018;47:1623-35.

[4] Morris JS. Functional regression. Annual Review of Statistics and Its Application. 2015;2:321-59.

[5] Kokoszka P, Reimherr M. Introduction to functional data analysis. CRC press; 2017.

[6] Shi JQ, Choi T. Gaussian process regression analysis for functional data. CRC press; 2011.

[7] Vehtari A, Gelman A, Simpson D, Carpenter B, Bürkner PC. Rank-normalization, folding, and localization: An improved Rhat for assessing convergence of MCMC (with discussion). Bayesian analysis. 2021;16:667-718.

[8] Gelman A, Carlin JB, Stern HS, Rubin DB. Bayesian data analysis. Chapman and Hall/CRC; 1995.

[9] Modrák M, Moon AH, Kim S, Bürkner P, Huurre N, Faltejsková K, et al. Simulation-based calibration checking for Bayesian computation: The choice of test quantities shapes sensitivity. arXiv preprint arXiv:221102383. 2022.

[10] Talts S, Betancourt M, Simpson D, Vehtari A, Gelman A. Validating Bayesian inference algorithms with simulation-based calibration. arXiv preprint arXiv:180406788. 2018.

[11] Kim S, Moon H, Modrák M, Säilynoja T. SBC: Simulation Based Calibration for rstan/cmdstanr models; 2023. Https://hyunjimoon.github.io/SBC/, https://github.com/hyunjimoon/SBC/.
